# La leptina promueve la expresión de Hic-5 y la formación de puntos de actina por la vía dependiente de FAK-Src en células epiteliales mamarias MCF10A

**DOI:** 10.7705/biomedica.4313

**Published:** 2019-09-01

**Authors:** Raúl Isaías-Tizapa, Erika Acosta, Arvey Tacuba-Saavedra, Miguel Mendoza-Catalán, Napoleón Navarro-Tito

**Affiliations:** 1 Facultad de Ciencias Químico-Biológicas, Universidad Autónoma de Guerrero, Chilpancingo de los Bravo, México Universidad Autónoma de Guerrero Facultad de Ciencias Químico-Biológicas Universidad Autónoma de Guerrero Chilpancingo de los Bravo Mexico

**Keywords:** leptina, neoplasias, metástasis de la neoplasia, actinas, transición de epitelio a mesénquima, Leptin, neoplasms, neoplasm metastasis, actins, epithelial-mesenchymal transition

## Abstract

**Introducción.:**

La leptina es una hormona secretada por los adipocitos que se ha relacionado con el proceso de la transición de epitelio a mesénquima (*Epithelial- Mesenchymal Transition*, EMT). Promueve la migración e invasión de las células del epitelio mamario mediante la activación de las cinasas FAK y Src, un complejo regulador de vías de señalización que favorecen la expresión de las proteínas relacionadas con la formación de estructuras proteolíticas implicadas en la invasión y progresión del cáncer. Recientemente, se ha descrito que la sobreexpresión y activación de la proteína Hic-5 durante el mencionado proceso de transición, favorece la formación de los puntos de actina (indicativa de la formación y funcionalidad de los invadopodios), lo cual promueve la degradación local de los componentes de la matriz extracelular y la metástasis del cáncer.

**Objetivos.:**

Evaluar el papel de las cinasas FAK y Src sobre la expresión y localización subcelular de Hic-5 y la formación de puntos de actina inducida por la leptina en la línea celular MCF10A de epitelio mamario no tumoral.

**Materiales y métodos.:**

Se utilizaron los inhibidores específicos de la FAK (PF-573228) y la Src (PP2) para evaluar el papel de ambas cinasas en los niveles de expresión y localización subcelular de la proteína Hic-5 mediante *Western blot* e inmunofluorescencia, así como la formación de puntos de actina mediante la tinción con faloidina-TRITC en células MCF10A estimuladas con leptina.

**Resultados.:**

La leptina indujo el incremento en la expresión de Hic-5 y la formación de puntos de actina. El tratamiento previo con los inhibidores de las cinasas FAK (PF-573228) y Src (PP2), promovió la disminución en la expresión de Hic-5 y de los puntos de actina en la línea celular MCF10A de epitelio mamario no tumoral.

**Conclusión.:**

La leptina indujo la expresión y la localización perinuclear de Hic-5 y la formación de puntos de actina mediante un mecanismo dependiente de la actividad de las cinasas FAK y Src en las células MCF10A.

## Abstract (en)

**Introduction:**

Leptin is a hormone secreted by adipocytes that has been associated with the epithelial-mesenchymal transition (EMT). Additionally, leptin promotes the migration and invasion of mammary epithelial cells through the activation of FAK and Src kinases, which are part of a regulatory complex of signaling pathways that promotes the expression of proteins related to the formation of proteolytic structures involved in the invasion and progression of cancer. Recently, overexpression and activation of Hic-5 during the EMT have been shown to induce the formation of actin puncta; these structures are indicative of the formation and functionality of invadopodia, which promote the local degradation of extracellular matrix components and cancer metastasis.

**Objective:**

To evaluate the role of FAK and Src kinases in the expression of Hic-5 during the epithelial-mesenchymal transition induced by leptin in MCF10A cells.

**Materials and methods:**

We used specific inhibitors of FAK (PF-573228) and Src (PP2) to evaluate Hic-5 expression and subcellular localization by Western blot and immunofluorescence assays and to investigate the formation of actin puncta by epifluorescence in MCF10A cells stimulated with leptin.

**Results:**

Leptin induced an increase in Hic-5 expression and the formation of actin puncta. Pretreatment with inhibitors of FAK (PF-573228) and Src (PP2) promoted a decrease in Hic-5 expression and actin puncta formation in the non-tumorigenic mammary epithelial cell line MCF10A.

**Conclusion:**

In MCF10A cells, leptin-induced Hic-5 expression and perinuclear localization, as well as the formation of actin puncta through a mechanism dependent on the kinase activity of FAK and Src.

La leptina es una hormona codificada por el gen *LEP* en humanos y es secretada en la placenta y los ovarios. Su función primordial en el tejido adiposo es regular la saciedad y el gasto energético, actuando directamente en el hipotálamo ^1^^,^^2^. Los niveles séricos de leptina en personas con peso normal (índice de masa corporal, IMC, de 18,5 a 24,9 kg/m2) se encuentran en un rango entre 5 y 50 ng/ml, en tanto que, en personas obesas, los niveles superan los 100 ng/ml ^3^.

La leptina está implicada en la regulación de procesos fisiológicos y, recientemente, se la ha asociado con la progresión tumoral del cáncer de mama, la cual se caracteriza por la proliferación acelerada y descontrolada de las células del epitelio glandular mamario ^4^. Este tipo de cáncer es el más común entre las mujeres a nivel mundial. En Latinoamérica, la tasa de incidencia del cáncer de mama es de 100.000 casos anuales, aproximadamente, siendo la causa de muerte más importante por tumores malignos en las mujeres, incluso, más que el cáncer de cuello uterino, con una tasa de mortalidad del 18 % del total de las defunciones por tumores malignos y del 3 % del total de las muertes en mujeres ^5^^,^^6^.

Yuan, *et al.,* han reportado que la leptina indujo la proliferación y la migración de la línea celular MCF-7 de cáncer mamario y han sugerido que es una hormona capaz de inducir cambios en la proliferación, la supervivencia y el crecimiento, y que promueve la invasión y la metástasis de las células tumorales ^7^. Por otra parte, estos autores describieron cómo el tratamiento con leptina en líneas celulares de cáncer de mama indujo el proceso de transición de epitelio a mesénquima (*Epithelial-Mesenchymal Transition*, EMT), proceso biológico relacionado con la progresión tumoral, y con un fenotipo invasivo y metastásico ^8^.

La transición de epitelio a mesénquima ocurre cuando las células epiteliales pierden su polaridad apical-basolateral, presentan una disminución en las uniones adherentes entre las células y entre estas y la matriz extracelular (transición e epitelio a mesénquima), adquiriendo características mesenquimatosas, entre estas, una morfología similar a los fibroblastos, resistencia a la apoptosis y mayor capacidad migratoria e invasiva ^9^^,^^10^.

A nivel molecular, se ha registrado disminución en la expresión de los marcadores epiteliales, como las proteínas cadherina-E, citoqueratinas y ocludinas, así como aumento en la expresión de marcadores mesenquimatosos como cadherina-N, vimentina y fibronectina.

Inicialmente, la transición de epitelio a mesénquima se asoció con procesos no patológicos, como la embriogénesis y la regeneración de tejidos; sin embargo, también puede ocurrir en eventos patológicos como la progresión tumoral ^11^^,^^12^. Se han descrito diversos inductores de dicha transición, entre ellos, el factor transformador de crecimiento β (*Transforming Growth Factor*, TGF-β), el factor de crecimiento epidérmico (*Epidermal Growth Factor*, EGF) y factores de transcripción como el Snail, el Slug y el Twist; recientemente, la leptina también se ha asociado como inductora de este programa ^13^.

Se ha establecido que la leptina ejerce su acción biológica mediante la unión a su receptor ObR, al promover la activación de diversas vías de señalización, como la vía PI-3K/Akt, que se asocia con el crecimiento y la supervivencia celular; la vía JAK/STAT3, que participa en la transcripción del gen del factor de crecimiento del endotelio vascular (*Vascular Endothelial Growth Factor*, VEGF) favorecedor de la angiogénesis; la vía MAPK (*Mitogen-Activated Protein Kinase*), que promueve la proliferación celular mediante la inactivación del inhibidor del ciclo celular pRb, y la vía PI-3K/Rac, la cual está implicada en la migración e invasión celular y, en consecuencia, en la progresión del cáncer ^14^^,^^15^.

Uno de los eventos asociados con la invasión celular es la formación de ‘invadopodios’, pequeñas protuberancias ricas en actina F organizadas en forma de puntos, las cuales se localizan en el frente invasivo de las células tumorales que degradan los componentes de la matriz extracelular de manera local. La formación de invadopodios se caracteriza por el reclutamiento y la activación de múltiples proteínas que regulan la polimerización de la actina, como la N-WASP, la Arp 2/3 y la cortactina, proteínas de señalización como la FAK y la Src, y proteínas adaptadoras, como la Hic-5, que tiene un peso molecular de 55 kDa y es codificada por el gen *TGFB1I1*.

En los estudios recientes se ha evidenciado la sobreexpresión y la activación de la Hic-5 durante la formación de los puntos de actina como parte de la formación de los invadopodios ^10^^,^^16^^,^^17^. La Hic-5 está constituida por un dominio N-terminal que contiene cuatro regiones ricas en leucina y aspartato (LD 1-4), y el dominio C-terminal, con cuatro regiones ricas en cisteína (LIM 1-4) ^18^^,^^19^. Los dominios LD y LIM regulan la interacción entre proteínas con diversas moléculas de señalización, como la FAK, promoviendo la formación de las adhesiones focales ^20^.

La FAK es una cinasa de tirosina con un peso molecular de 125 kDa que se activa mediante interacciones con las integrinas, los receptores de cinasa de tirosina, los receptores de citocinas y los receptores acoplados a proteínas G ^21^. La activación de la FAK se inicia con la autofosforilación en la tirosina 397, creando un sitio de unión para el dominio SH2 de la cinasa Src al promover su autofosforilación en la tirosina 419 y, en consecuencia, su activación.

La interacción entre la FAK y la Src permite que esta última fosforile las tirosinas 576 y 577 localizadas en el dominio cinasa de la FAK y promueva su máxima actividad catalítica. El complejo de cinasas FAK-Src regula importantes procesos celulares, como la adhesión, la motilidad, la proliferación y la supervivencia celular ^22^^-^^24^.

Recientemente, se ha reportado en modelos *in vitro* que la sobreexpresión, la localización subcelular y la activación de la proteína Hic-5 dependen de la actividad de las cinasas FAK y Src. Este evento es necesario para la formación y la funcionalidad de los invadopodios, los cuales están asociados con procesos invasivos durante la transición de epitelio a mesénquima inducida por el TGF-β en las células MCF10A ^17^^,^^25^.

En un estudio anterior, se reportó que la leptina inducía la activación de las cinasas FAK y Src en células MFC10A y en células cancerosas mamarias (Juárez-Cruz JC, García-Rodríguez E, Castañeda-Saucedo E, Mendoza- Catalán MA, Villegas S, *et al.* Leptin induces cell migration, gelatinases secretion and invasion in a FAK-Src dependent pathway in breast cancer cell. 2018. En prensa) ^26^. Sin embargo, no se ha estudiado la asociación existente entre la actividad de las cinasas FAK y Src y la expresión y la localización subcelular de la Hic-5 y la participación de esta en la formación de puntos de actina durante la transición de epitelio a mesénquima inducida por la leptina en células MCF10A del epitelio mamario no tumoral.

En este trabajo se evaluaron el efecto directo de la leptina y el papel de las cinasas FAK y Src sobre los niveles de expresión de la Hic-5 y la formación de estructuras de invasión local en células epiteliales mamarias MCF10A no tumorales.

## Materiales y métodos

### Anticuerpos y reactivos

La leptina recombinante humana, el EGF, la hidrocortisona, el medio DMEM/F12, el Fluoroshield/DAPI™, los inhibidores de FAK (PF-573228) y el inhibidor de Src (PP2), se obtuvieron de Sigma-Aldrich (Saint Louis, MO, USA). Los anticuerpos primarios anti-Hic-5, anti-actina β y los anticuerpos secundarios antirratón y anticonejo, se obtuvieron de Santa Cruz Biotechnology, Inc. (Santa Cruz, CA). El anticuerpo secundario Alexa Flour 488™ se obtuvo de Molecular Probes by Life Technologies (Eugene, Oregon, USA), la faloidina-TRITC™, de Thermo Fisher (Waltham, MA), el anticuerpo anti-GAPDH™, de Cell Signaling Technology (Danvers, MA), la insulina, de Humulin, y el suero fetal bovino (SFB), de By Productos (Guadalajara, MX).

### Cultivo celular

Las líneas celulares MCF10A y MDA-MB-231 se obtuvieron de la *American Type Culture Collection*, ATCC. La línea celular MCF10A derivada de epitelio mamario no tumoral, se cultivó en medio DMEM/ F12 con suplemento de 10 % de SFB, 10 µg/ml de insulina, 0,5 µg/ml de hidrocortisona, 20 ng/ml de EGF y 1 % de antibióticos (penicilina G y estreptomicina), en una atmósfera humidificada con 5 % de CO2 a temperatura constante de 37 °C.

Para los fines experimentales, en los cultivos confluentes se suprimieron el SFB y los utilizados durante cuatro horas antes del tratamiento con leptina y con los suplementos químicos de la FAK y la Src. La línea celular MDA- MB-231 se sembró en cubreobjetos y se mantuvo con medio DMEM/F12 y suero fetal bovino al 5 % hasta el momento de la inmunofluorescencia.

### Protocolo de estimulación

Para la técnica de inmunofluorescencia, los cultivos celulares se sembraron sobre cubreobjetos en placas de 24 pozos hasta adquirir el 60 % de confluencia y, para la técnica *Western blot*, los cultivos se mantuvieron en cajas de 60 mm hasta adquirir una confluencia del 80 al 90 %. En los cultivos celulares se suprimió el SFB con medio basal (DMEM/F12) durante cuatro horas y luego se trataron con los inhibidores selectivos de la FAK (PF-573228, 10 µM) y de la Src (PP2, 10 µM), ambos disueltos en DMSO, 30 minutos antes del estímulo durante 24 horas con leptina (400 ng/ml); la estimulación celular se concluyó con la remoción del medio. Los controles se trataron con el vehículo de leptina (Tris-HCl, pH 7,4).

Para los ensayos de formación de puntos de actina, se hizo la supresión en los cultivos confluentes y, luego, se estimularon y se cosecharon; posteriormente, las células se sembraron durante seis horas en cubreobjetos recubiertos con una matriz de gelatina bovina. Todos los experimentos se hicieron por triplicado y en momentos independientes.

### Western blot

Para la extracción de las proteínas totales, las células se diluyeron en 0,5 ml de solución tampón RIPA, con 50 mM de HEPES (Sigma-Aldrich), pH 7,4, 150 mM de NaCl, 1 mM de ácido egtázico (EGTA, Sigma-Aldrich), 1 mM de ortovanadato de sodio, 100 mM de NaF, 10 mM de pirofosfato de sodio, 10 % de glicerol, 1 % de tritón X-100, 1 % de deoxicolato de sodio, 1,5 Mm de MgCl2, 0,1 % de dodecilsulfato sódico (SDS), y 1 mM de fluoruro de fenilmetilsulfonilo (PMSF).

Se colocaron 20 µg de proteínas totales de cada condición y se separaron mediante electroforesis en gel de poliacrilamida con dodecil sulfato de sodio (*Sodium Dodecyl Sulfate Polyacrylamide Gel Electrophoresis,* SDS-PAGE) para, después, transferirlas a una membrana de nitrocelulosa.

La expresión de la proteína Hic-5 se determinó incubando la membrana con el anticuerpo primario anti-Hic-5 (dilución 1:500) y usando como controles de carga la actina β (dilución de 1:1000), incubada durante toda la noche a 4 °C, y la gliceraldehído-3-fosfato deshidrogenasa (GAPDH) en dilución de 1:500, durante cuatro horas a temperatura ambiente. Terminado el tiempo de incubación, las membranas se lavaron y se incubaron con el anticuerpo secundario anticonejo y antirratón durante dos horas a temperatura ambiente.

Por último, la inmunodetección se hizo con el kit Immun-Star WesternC™ (BIO-RAD) y placas autorradiográficas. La intensidad relativa se determinó mediante un análisis densitométrico de las bandas con el programa Image J, versión 1,44p.

### Inmunofluorescencia

Los cubreobjetos se dejaron toda la noche en ácido sulfúrico al 20 %; después, se esterilizaron con etanol al 96 % por dos horas. Posteriormente, los cultivos de las células MCF10A se sembraron sobre los cubreobjetos durante 48 horas hasta alcanzar una confluencia del 60 % y, enseguida, se aplicó el protocolo de estimulación durante 24 horas con las diferentes condiciones experimentales.

Al finalizar el tratamiento de las células MCF10A y las células MDA- MB-231 de cáncer de mama sin tratar, estas se fijaron y permeabilizaron con formaldehído al 4 % y tritón X-100 al 0,5 % en PBS y se bloquearon con albúmina de suero bovino (*Bovine Serum Albumin,* BSA) al 3 %. Se incubaron con el anticuerpo primario anti-Hic-5 en una dilución de 1:100 durante dos horas a temperatura ambiente y el anticuerpo secundario antirratón marcado con Alexa Fluor 488™ en una dilución de 1:400 durante 30 minutos a 37 oC, se montaron en los cubreobjetos en Fluoroshield™/DAPI y, por último, se observaron bajo el microscopio de epifluorescencia Olympus BX43™ con el objetivo de 100X.

Ensayo de formación de puntos de actina

Los cultivos confluentes fueron estimulados bajo las condiciones experimentales descritas en el protocolo de estimulación; al terminar, se cosecharon, se sembraron durante seis horas sobre una matriz de gelatina bovina no fluorescente. A las 6 horas, las células se fijaron y permeabilizaron con formaldehído al 4 % y tritón X-100 al 0,5 % en PBS, y se bloquearon con BSA al 3 %. Se incubaron con faloidina-TRITC 1:500 durante 30 minutos a 37 oC, se montaron en los cubreobjetos en Fluoroshield™/DAPI y, finalmente, se observaron bajo el microscopio de epifluorescencia Olympus BX43™ utilizando el objetivo de 100X.

### Análisis estadístico

Los datos se examinaron mediante la prueba t de Student y el análisis de varianza (ANOVA) de una vía con la prueba de comparación múltiple de Newman-Keuls, y los resultados se expresaron como media ± desviación estándar (DE), tomando como estadísticamente significativo un valor de p<0,05. Por último, los resultados de los ensayos se graficaron con el programa estadístico GraphPad Prism 5,0.

## Resultados

**La leptina promovió la expresión de la Hic-5 en la línea celular MCF10A*.***

Para determinar el efecto de la leptina en la expresión de la proteína Hic- 5, se utilizó la prueba *Western blot* a partir de los extractos de proteínas de cultivos de células MCF10A estimuladas con 400 ng/ml de leptina durante 24 horas, y se utilizó como control el vehículo de leptina (Tris-HCl).

**Figura 1:**
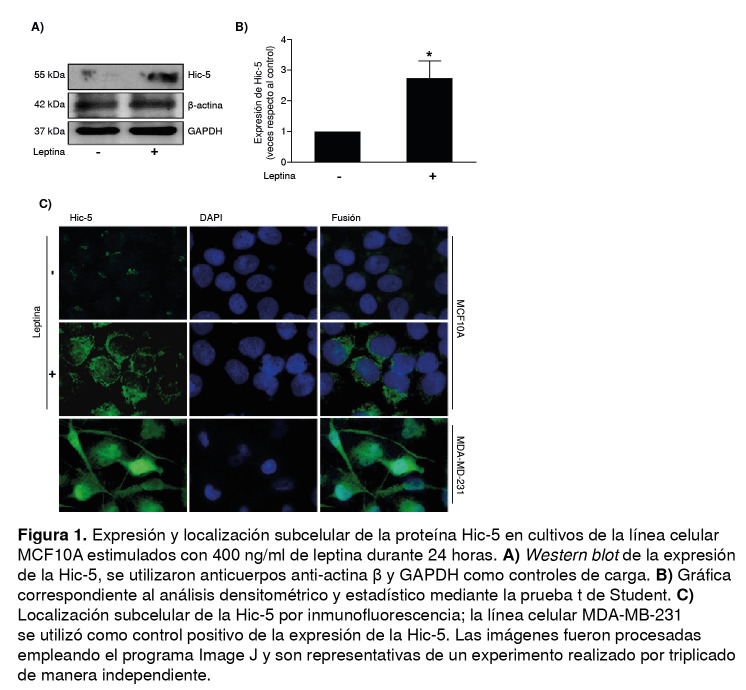
Expresión y localización subcelular de la proteína Hic-5 en cultivos de la línea celular MCF10A estimulados con 400 ng/ml de leptina durante 24 horas. **A)** *Western blot* de la expresión de la Hic-5, se utilizaron anticuerpos anti-actina β y GAPDH como controles de carga. **B)** Gráfica correspondiente al análisis densitométrico y estadístico mediante la prueba t de Student. **C)** Localización subcelular de la Hic-5 por inmunofluorescencia; la línea celular MDA-MB-231 se utilizó como control positivo de la expresión de la Hic-5. Las imágenes fueron procesadas empleando el programa Image J y son representativas de un experimento realizado por triplicado de manera independiente.

Los resultados demostraron que la leptina indujo un incremento de la expresión de la Hic-5 en la línea celular MCF10A de epitelio mamario no tumoral, comparada con el control (figura 1 A y B). La localización subcelular de la Hic-5 se evaluó mediante inmunofluorescencia, y se observó una mayor expresión y distribución perinuclear de la Hic-5 en los cultivos estimulados con leptina que en las células no estimuladas. Como control positivo de la expresión de la Hic-5, se emplearon cultivos de células MDA-MB-231 de cáncer mamario (figura 1C).

### La leptina indujo la expresión de la Hic-5 mediante la actividad cinasa de la FAK en la línea celular MCF10A.

Para determinar si la leptina inducía la expresión de la proteína Hic- 5 mediante la actividad cinasa de la FAK en la línea celular MCF10A, los cultivos celulares se sometieron a supresión con medio basal durante cuatro horas y se trataron con el inhibidor de la FAK (PF-573228) 30 minutos antes de estimularlos con 400 ng/ml de leptina durante 24 horas.

**Figura 2:**
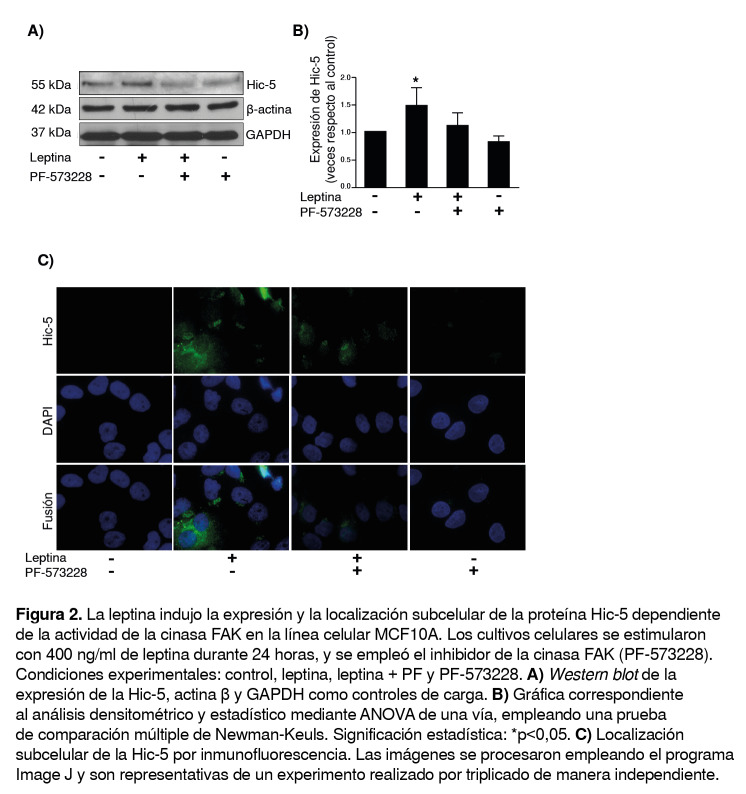
La leptina indujo la expresión y la localización subcelular de la proteína Hic-5 dependiente de la actividad de la cinasa FAK en la línea celular MCF10A. Los cultivos celulares se estimularon con 400 ng/ml de leptina durante 24 horas, y se empleó el inhibidor de la cinasa FAK (PF-573228). Condiciones experimentales: control, leptina, leptina + PF y PF-573228. **A)** **Western blot** de la expresión de la Hic-5, actina β y GAPDH como controles de carga. **B)** Gráfica correspondiente al análisis densitométrico y estadístico mediante ANOVA de una vía, empleando una prueba de comparación múltiple de Newman-Keuls. Significación estadística: *p<0,05. **C)** Localización subcelular de la Hic-5 por inmunofluorescencia. Las imágenes se procesaron empleando el programa Image J y son representativas de un experimento realizado por triplicado de manera independiente.

Se extrajeron las proteínas totales y se hicieron las pruebas *Western blot* utilizando un anticuerpo específico para la Hic-5. En este modelo experimental, los resultados mostraron una mayor expresión de la Hic-5 con leptina comparada con el control; sin embargo, al agregar el tratamiento previo con el inhibidor de FAK más leptina, la expresión de la Hic-5 disminuyó, comparada con la leptina sola (figura 2 A, B).

Además, en este estudio se observó una mayor expresión y localización perinuclear de la Hic-5 bajo el estímulo con leptina que con el control. Sin embargo, con el inhibidor de la FAK y el estímulo con leptina, la expresión de la Hic-5 disminuyó en comparación con el estímulo de la leptina únicamente (figura 2 C). Estos resultados reflejan que la expresión y la localización perinuclear de la Hic-5 ocurren mediante un mecanismo que depende de la actividad cinasa de la FAK.

### La leptina indujo la expresión de la Hic-5 mediante la actividad cinasa de la Src en la línea celular MCF10A.

**Figura 3:**
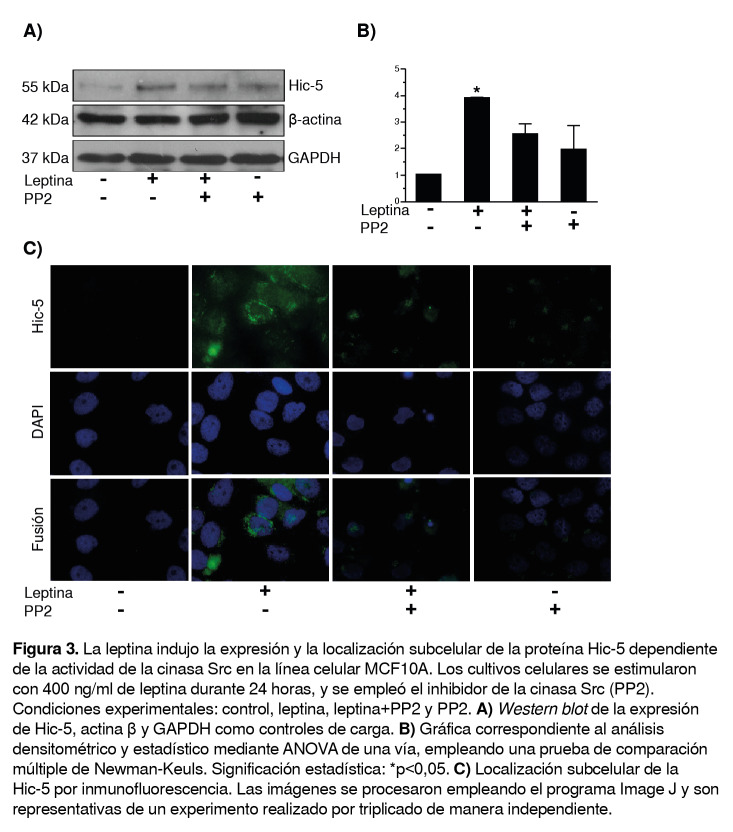
La leptina indujo la expresión y la localización subcelular de la proteína Hic-5 dependiente de la actividad de la cinasa Src en la línea celular MCF10A. Los cultivos celulares se estimularon con 400 ng/ml de leptina durante 24 horas, y se empleó el inhibidor de la cinasa Src (PP2). Condiciones experimentales: control, leptina, leptina+PP2 y PP2. **A)** *Western blot* de la expresión de Hic-5, actina β y GAPDH como controles de carga. **B)** Gráfica correspondiente al análisis densitométrico y estadístico mediante ANOVA de una vía, empleando una prueba de comparación múltiple de Newman-Keuls. Significación estadística: *p<0,05. **C)** Localización subcelular de la Hic-5 por inmunofluorescencia. Las imágenes se procesaron empleando el programa Image J y son representativas de un experimento realizado por triplicado de manera independiente.

En cuanto al papel de la cinasa Src en la expresión y localización celular de la Hic-5, se observó un incremento en la expresión de esta proteína al emplear leptina comparada con el control; sin embargo, al agregar el tratamiento previo con el inhibidor de la Src y el estímulo con leptina, la expresión de la Hic-5 disminuyó en comparación con el estímulo de leptina únicamente (figura 3A, B).

Tanto en la condición con el inhibidor de la FAK como con el de la Src, con y sin tratamiento con leptina, la expresión y la localización perinuclear de la Hic-5 disminuyeron comparadas con el estímulo con leptina (figura 3C), lo cual sugiere que Hic-5 se produce mediante un mecanismo que depende de la actividad de cinasa de Src y de FAK.

### La leptina indujo la formación de puntos de actina de manera dependiente de la actividad cinasa de la FAK y la Src en la línea celular MCF10A.

Para determinar si la leptina promovía la formación de puntos de actina en la línea celular MCF10A, los cultivos celulares se estimularon con 400 ng/ml de leptina durante 24 horas, y posteriormente, se cosecharon y sembraron en una matriz de gelatina bovina no fluorescente durante seis horas, con lo que se observaron estructuras densas de F-actina en forma de puntos, sugestivas de la formación de invadopodios como reacción a la estimulación con leptina.

El efecto de la actividad de las cinasas FAK y Src se evaluó mediante cultivos tratados previamente con los inhibidores de la FAK (PF-573228) y la Src (PP2) y posteriormente estimulados con 400 ng/ml de leptina durante 24 horas.

**Figura 4:**
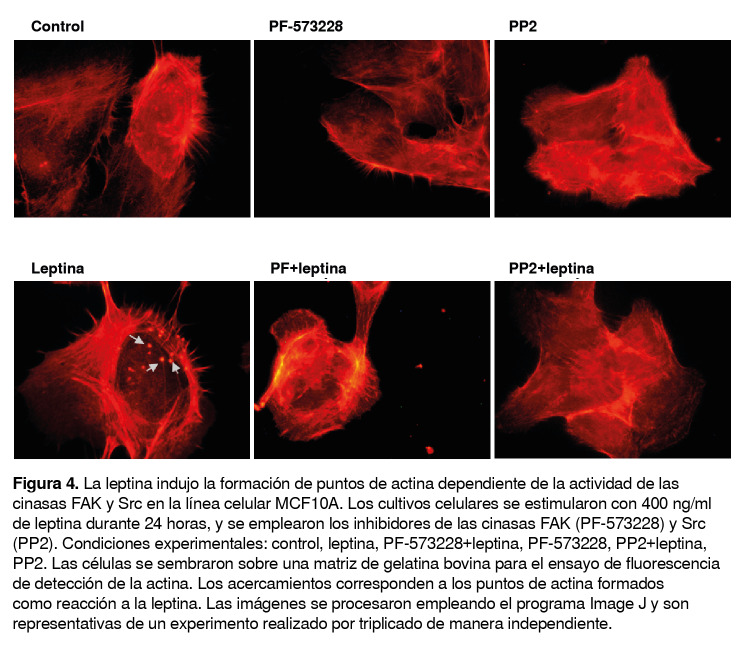
La leptina indujo la formación de puntos de actina dependiente de la actividad de las cinasas FAK y Src en la línea celular MCF10A. Los cultivos celulares se estimularon con 400 ng/ml de leptina durante 24 horas, y se emplearon los inhibidores de las cinasas FAK (PF-573228) y Src (PP2). Condiciones experimentales: control, leptina, PF-573228+leptina, PF-573228, PP2+leptina, PP2. Las células se sembraron sobre una matriz de gelatina bovina para el ensayo de fluorescencia de detección de la actina. Los acercamientos corresponden a los puntos de actina formados como reacción a la leptina. Las imágenes se procesaron empleando el programa Image J y son representativas de un experimento realizado por triplicado de manera independiente.

Los datos obtenidos evidenciaron que la formación de puntos de actina como reacción a la leptina fue dependiente de la actividad de las cinasas FAK y Src (figura 4). Llamó la atención la formación de fibras de estrés en las células estimuladas con leptina, en comparación con las células estimuladas con el vehículo de leptina. Además, se observó la disminución de estas fibras en presencia de los inhibidores de las cinasas FAK y Src (figura 4).

## Discusión

En diversos estudios clínicos y epidemiológicos, se ha determinado que las altas concentraciones de leptina en el suero se relacionan con un mayor riesgo de desarrollar cáncer de mama en mujeres antes y después de la menopausia ^27^.

Los datos publicados por Garofalo, *et al.,* demostraron que tanto la leptina como su receptor ObR se encuentran sobreexpresados en el 83 % de los tumores de cáncer mamario en estadio primario y metastásico, comparados con tejido no canceroso ^28^. En modelos *in vitro* se ha descrito que la leptina actúa como inductor del proceso de la transición de epitelio a mesénquima y la activación de las cinasas FAK y Src (Juárez-Cruz JC, García-Rodríguez E, Castañeda-Saucedo E, Mendoza-Catalán MA, Villegas S, *et al*. Leptin induces cell migration, gelatinases secretion and invasion in a FAK-Src dependent pathway in breast cancer cell. 2018. En prensa) ^26^. Pignatelli, *et al.,* demostraron que la actividad de estas cinasas promueve la activación de la Hic-5, una proteína adaptadora que participa en las adhesiones focales y que en los últimos años se ha asociado con la progresión tumoral ^17^.

En el presente estudio se observó que la leptina promovió la expresión y la localización perinuclear de la Hic-5 en células MCF10A del epitelio mamario no tumoral (figura 1). En este sentido, en estudios de pacientes con cáncer de ovario e hígado, se ha establecido que la Hic-5 se encuentra sobreexpresada en el tejido canceroso en comparación con el tejido sano; además, en líneas celulares de cáncer de mama se ha reportado la sobreexpresión de esta proteína y se ha asociado con procesos que favorecen la progresión del cáncer ^29^^-^^31^.

Los datos publicados por Sheta, *et al.,* demostraron que la Hic-5 regulaba la transición de epitelio a mesénquima mediante un mecanismo independiente del TGFβ1 en células de cáncer de ovario ^30^. Asimismo, Pignatelli, *et al.,* demostraron que la invasión inducida por el TGFβ en células MCF10A requiere de la activación de la Hic-5 mediante la cinasa Src, y reportaron que la Hic-5 activa participa en la formación de invadopodios, estructuras ricas en actina especializadas en la degradación de los componentes de dicha transición y que promueven la migración y la invasión celulares ^17^.

Se ha reportado que la activación de la FAK regula procesos celulares como la angiogénesis, la transición de epitelio a mesénquima y la metástasis de células cancerosas, al asociarse con la progresión a un fenotipo más agresivo en tumores mamarios ^32^. Además, un análisis con las pruebas de *Northern* y *Western blot* demostró que los niveles del ARNm y la proteína de la FAK se encontraban elevados en las muestras de los tumores de mama invasivos y metastásicos, en comparación con los tejidos normales, lo que sugiere la participación activa de la FAK en la invasión y metástasis en el cáncer de mama ^33^.

Por este motivo, en el presente estudio se decidió evaluar el papel de la FAK en los niveles de expresión de la Hic-5 en células tratadas con leptina. Los resultados obtenidos demostraron que la leptina promueve la expresión de la Hic-5 y que ello depende de la actividad cinasa de la FAK (figura 2A, B). Los resultados que relacionan la participación del complejo Src-FAK con la expresión de la Hic-5 explicarían la activación de la Src cuando se une a la tirosina 397 fosforilada de la FAK; una vez activa, la Src promueve la fosforilación de la tirosina 925 de la FAK, lo cual genera un sitio de interacción con la proteína adaptadora Grb2 y, subsecuentemente, la activación de la vía de señalización de la MAPK ^34^.

Por otro lado, se ha reportado que la asociación de la FAK con la Src causa la fosforilación en p130Cas y el reclutamiento de Crk ^35^. Este complejo permite, a su vez, la activación de la JNK, una proteína relacionada con la activación del factor de transcripción c-Jun que, según observaciones, promueve la expresión de la Hic-5 ^31^. En este contexto, se ha propuesto la FAK como un importante biomarcador para la carcinogénesis y la progresión del cáncer mamario ^36^. Asimismo, se ha descrito que la leptina induce la migración celular en dos líneas de cáncer de mama: la MDA-MB-231, con fenotipo invasivo, y la línea no invasiva MCF7 (Juárez-Cruz JC, García- Rodríguez E, Castañeda-Saucedo E, Mendoza-Catalán MA, Villegas S, *et al*. Leptin induces cell migration, gelatinases secretion and invasion in a FAK-Src dependent pathway in breast cancer cell. 2018. En prensa). También, se ha descrito que la leptina promueve la fosforilación y la activación de la FAK y la ERK, proteínas relacionadas con la transición de epitelio a mesénquima en las células MCF10A. Estos datos sugieren que la expresión de la Hic-5 está relacionada con una vía dependiente de las cinasas Src-FAK y ERK en la línea celular MCF10A ^26^.

La Src regula múltiples procesos celulares relacionados con la progresión tumoral, como la angiogénesis, la adhesión, la motilidad, la invasión y la migración celular, lo cual sugiere que la Src tiene un papel importante en el desarrollo del tumor y la metástasis ^37^^-^^39^. Verbeek, et *al*., encontraron que en las muestras de tejido de cáncer de mama había un aumento en la expresión y la actividad de la Src, comparadas con las del tejido mamario normal ^40^. Además, en los estudios de pacientes con cáncer de mama, se ha descrito que el aumento de la expresión y la actividad de la Src reducía considerablemente su tasa de supervivencia ^41^^,^^42^.

Por esta razón, en el presente estudio, se evaluó el efecto de la cinasa Src sobre los niveles de expresión de la Hic-5 en células tratadas con leptina. Resultó interesante observar que la expresión de Hic-5 aumentaba con el tratamiento con leptina, sin embargo, disminuyó tras la inhibición química de la Src (figura 3A y 3B).

Este evento puede ocurrir por la activación del receptor de la leptina durante el tratamiento, lo que favorece su fosforilación en los sitios de las tirosinas 985, 1077 y 1138 ^43^. La Src posee un dominio SH2 en su extremo N-terminal, el cual reconoce tirosinas fosforiladas y puede unirse a la caja 1 del receptor de leptina y, en consecuencia, promover su activación; cuando esto ocurre, se puede inducir la activación de la cinasa FAK fosforilando los residuos de las tirosinas 576 y 577 localizados en el dominio cinasa de esta proteína y ocasionando su máxima actividad catalítica ^44^. Cuando la Src no se activa se interrumpen todos los eventos moleculares regulados por dicha activación, en este caso particular, la expresión de la Hic-5 inducida por la leptina en las células MCF10A del epitelio mamario.

La función de las proteínas en las células se relaciona, en parte, con su localización subcelular. Para determinar la localización subcelular de la Hic-5, en el presente estudio se hicieron pruebas de inmunofluorescencia cuyos resultados evidenciaron una mayor expresión bajo el estímulo con leptina y una distribución perinuclear de esta proteína. En la figura 2C se observa una mayor expresión y localización perinuclear de la Hic-5 con leptina, en comparación con el control; sin embargo, con el inhibidor de la FAK y el estímulo con leptina, la expresión y la localización perinuclear de la Hic-5 disminuyeron en comparación con lo que sucede con el estímulo con leptina. Además, con el inhibidor de la Src más el estímulo con leptina, la expresión y la localización perinuclear de la Hic-5 disminuyeron en comparación lo que sucede con el estímulo con leptina (figura 3C).

Cabe resaltar que la localización perinuclear de la Hic-5 podría estar relacionada con un marcador de la transición de epitelio a mesénquima y la formación de invadopodios. Pignatelli, *et al.,* demostraron que el TGF-β promueve la formación de invadopodios y un aumento en la expresión de la Hic-5 ^17^. Curiosamente, la inhibición de la expresión de la Hic-5 disminuyó la degradación de la matriz extracelular, lo que sugiere que es responsable de la degradación de la transición de epitelio a mesénquima inducida por el TGF-β.

Por otro lado, se ha determinado que la FAK se encuentra activa en los invadopodios y que su sobreexpresión promueve la degradación de la matriz extracelular. Asimismo, la actividad de la Src es necesaria para la formación de invadopodios. Estos datos sugieren que la leptina promueve la expresión y la localización perinuclear de la Hic-5 y que ello podría relacionarse con un evento crucial durante la transición de epitelio a mesénquima consistente en la invasión local o formación de invadopodios ^17^.

En experimentos *in vitro* se ha demostrado que la forma activa de la FAK y la Src promueve la polimerización y reorganización de la actina, generando diferentes estructuras, incluidos los lamelopodios, las fibras de estrés y los puntos de actina, necesarios para la movilidad e invasión local de las células. Asimismo, Yamaguchi, *et al.,* asociaron la formación de estos puntos de actina con etapas iniciales de la formación de los invadopodios en células tumorales, lo que promueve la degradación de la matriz extracelular ^45^^-^^47^.

En el presente estudio, se confirmó que la leptina promueve la formación de puntos de actina por una vía dependiente de la FAK y la Src en células MCF10A, un evento biológico que permite la formación y la maduración de los invadopodios y, en consecuencia, la metástasis del cáncer (figura 4).

Al observar que la expresión de la Hic-5 inducida por la leptina se da parcialmente mediante un mecanismo dependiente del complejo de las cinasas FAK y Src, se propone que dicha expresión ocurre, en parte, mediante ambas cinasas y que otros mecanismos de señalización están involucrados con esta expresión. Además, se sugiere que la expresión de la Hic-5 se relaciona con procesos invasivos, como la formación de invadopodios y la posterior degradación de la matriz extracelular.

Los datos obtenidos en este estudio podrían estar relacionados con un evento biológico *in vivo* en el que el microambiente tumoral, específicamente la abundancia de leptina y su receptor, participan activamente en eventos relacionados con la transición de epitelio a mesénquima y, en consecuencia, con la invasión y metástasis de células tumorales en pacientes que presentan tanto obesidad como cáncer de mama.
